# Top-down, Bottom-up and Sideways: The Multilayered Complexities of Multi-level Actors Shaping Forest Governance and REDD+ Arrangements in Madre de Dios, Peru

**DOI:** 10.1007/s00267-017-0982-5

**Published:** 2018-01-03

**Authors:** Dawn Rodriguez-Ward, Anne M. Larson, Harold Gordillo Ruesta

**Affiliations:** 1 Center for International Forestry Research, Avenida La Molina 1895, La Molina, Lima, Peru; 2 NIRAS Finland Oy, Ratatie 11, Vantaa, Finland

**Keywords:** Multi-level forest governance, REDD+, Integrated landscape governance, Decentralization, Madre de Dios, Land use and land-use change

## Abstract

This study examines the role multilevel governance plays in the adoption of sustainable landscape management initiatives in emerging arrangements aimed at reducing emissions from deforestation and forest degradation (REDD+). It sheds light on the challenges these multiple layers of actors and interests encounter around such alternatives in a subnational jurisdiction. Through transcript analysis of 93 interviews with institutional actors in the region of Madre de Dios, Peru, particularly with regard to five sites of land-use change, we identified the multiple actors who are included and excluded in the decision-making process and uncovered their complex interactions in forest and landscape governance and REDD+ arrangements. Madre de Dios is a useful case for studying complex land-use dynamics, as it is home to multiple natural resources, a large mix of actors and interests, and a regional government that has recently experienced the reverberations of decentralization. Findings indicate that multiple actors shaped REDD+ to some extent, but REDD+ and its advocates were unable to shape land-use dynamics or landscape governance, at least in the short term. In the absence of strong and effective regional regulation for sustainable land use alternatives and the high value of gold on the international market, illegal gold mining proved to be a more profitable land-use choice. Although REDD+ created a new space for multilevel actor interaction and communication and new alliances to emerge, the study questions the prevailing REDD+ discourse suggesting that better coordination and cooperation will lead to integrated landscape solutions. For REDD+ to be able to play a role in integrated landscape governance, greater attention needs to be paid to grassroots actors, power and authority over territory and underlying interests and incentives for land-use change.

## Introduction

Reducing Emissions from Deforestation and Forest Degradation (REDD+) is a global initiative supported through the United Nations Framework Convention on Climate Change (UNFCCC) process to lower greenhouse gas emissions from forest loss and degradation, but for the purposes of this paper, it is emblematic of similar multiple—past and present—attempts to promote more sustainable, integrated and/or climate friendly land uses. REDD+, together with more recent calls for low emissions development, green economy, and sustainable futures, is particularly important in light of efforts to mitigate climate change under the Paris Agreement (2015).

REDD+ is inherently a multilevel, multisector process, aimed narrowly, on the one hand, at reducing carbon emissions (McDermott 2012) but broadly, on the other, at shaping environmental governance (Thompson et al. 2011) and transforming the trajectory of land use and land-use change (Brockhaus et al. 2014; Sanders et al. 2017). Although increasingly seen as central to climate change discussions, there is little understanding of what we mean by multilevel governance or how it is relevant to the implementation of policies or initiatives such as REDD+ on the ground. This study used the concept of multilevel governance to explore actor relations and land-use trajectories in a subnational jurisdiction and positions the results and trends in the broader debate of integrated landscape governance based on multi-stakeholder approaches.

In many countries, REDD+ initiatives began with parallel processes of pilot project implementation and national level readiness activities (Ravikumar et al. 2015a; Wertz-Kanounnikoff and Angelsen 2009). Madre de Dios represents a region that was ahead of the game in moving toward a subnational jurisdictional REDD+, through the coordination and collaboration of initiatives at the regional (jurisdictional government) level. In spite of their growing importance in climate mitigation and land-use policy, few studies examine REDD+ at the subnational jurisdictional scale (see Sanders et al. 2017; Fishbein and Lee 2015; Ravikumar et al. 2015a; Duchelle et al. 2014).

This article uses the case study of REDD+ in Madre de Dios, Peru, to explore the potential of initiatives aimed at altering “business-as-usual” development trajectories, such as REDD+ and others aimed at climate mitigation. Specifically, it examines how key actor types (the state at multiple levels, private sector and civil society), configurations and institutional arrangements are contributing to implementation; the challenges these REDD+ arrangements are facing; and how they are, or are not, reconfiguring the future of landscape governance and land-use and land-use change decisions. Madre de Dios is a particularly interesting region in which to carry out this research as important efforts were made early on to bring multiple pilot initiatives together, at least to collaborate, under a single regional jurisdiction led in part by the regional government (Che Piu and Menton 2013). A total of 15 projects were said to exist at the time this research began (MSAR personal communication 2013; Perez 2013). In addition, Madre de Dios has the advantage of having high value timber and non-timber forest products (NTFPs—particularly Brazil nuts), high levels of cultural and biological diversity with internationally recognized ecotourism potential, and a low population density. Nevertheless, the region as a whole represents some of the most challenging, and perhaps more realistic, kinds of problems that efforts to alter development trajectories will have to face to bring about meaningful change.

We found that involvement in REDD+ initiatives and working groups created a space for dialog between multiple types of actors that had not come together previously. These interactions also facilitated certain interregional alliances and international relationships relevant for the current debate on integrated landscape approaches based on multi-stakeholder and cross-sector collaboration and negotiation (Sayer et al. 2013; Milder et al. 2014; Ros-Tonen et al. 2014; Ravikumar et al. 2017). Our study suggests, however, that while these various actors shaped REDD+ in the region, and did improve some land management practices in specific locations, they were not able to shape land-use dynamics (or governance relations) more broadly, at least in the short term. We argue that the multilayered dynamics found in Madre de Dios represent deep-rooted differences of interest as well as distrust of actors’ motives that suggest that fundamental questions of power and authority must be addressed before multilevel and multisectoral collaboration in integrated landscape approaches can begin to bring about transformational change. Whereas multilevel governance theorists sometimes see a diminished role for the state in polycentric climate mitigation solutions, this article argues that the state, not only at national, but also at subnational level, has a central role to play.

In the following section “Multilevel governance and REDD+” we discuss the importance of using a multilevel governance approach to examine multilevel actor involvement in REDD+ (and integrated landscape governance more broadly) and how different actors can influence its trajectory. We then offer a concise synthesis on the current landscape and drivers of deforestation in Madre de Dios to better understand the context of the region. The next section describes the methodology used for field site selection and data collection and analysis. Our results section describes the emergence of regional REDD+ initiatives and working groups, the multilevel actors and their influence in shaping REDD+, challenges found in advancing REDD+, and coalitions that emerged. The discussion section proposes lessons for “integrated landscape governance” and the paper concludes with final thoughts on our findings.

## Multilevel Governance and REDD+

REDD+ was initially conceived as a national strategy to reduce carbon dioxide emissions, whereby payments, mainly through carbon markets with some fund-based support, would be made based on verified results. REDD+ was slated to begin with subnational projects, designed most likely by NGOs or the private sector, which were seen as experimental pilot initiatives while national governments developed the necessary institutions and expertize (Vatn and Angelson 2009). Others suggested that subnational governments were likely to have substantial roles to play (Larson and Ribot 2009). In fact, some projects have been implemented by state or municipal governments (e.g., Acre, Brazil; see also Ravikumar et al. 2015a). Today, REDD+ is moving toward the integration of these experiments into national strategies with increasing importance given to subnational jurisdictions in that process.

This study complements research on REDD+ at the national level (http://www.cifor.org/gcs/modules/redd-policies/) and on pilot projects (http://www.cifor.org/gcs/modules/REDD-subnational-initiatives; see also Sills et al. 2014). Subnational governments frequently play a key role in decisions, if not in design at least in implementation, relating to policies and projects that affect forests, land use and land-use changes.^1^Since the 1980s, many decentralization policies have transferred responsibilities to subnational governments at either regional or local level, though important powers over forests have often remained centralized (Ribot et al. 2006). In Peru, however, regional governments play key decision-making roles in some spheres—particularly the granting of both land titles and forest concessions^2^Regional governments develop land-use plans (such as the Economic Ecological Zoning Plan) for their jurisdictions under the auspices of the central government’s Ministry of Environment. The regional governments also grant artisanal and small-scale mining concessions while large-scale mining, petroleum and road infrastructure concessions are granted by central government offices. Maneuvering these responsibilities on the ground poses complex governance challenges, as there are overlapping responsibilities between levels and sectors of government, resulting overlaps in titles and concessions (see infographic on legal authority over land use in Madre de Dios^3^) and budget and capacity challenges (Wieland Fernandini and Sousa 2015).

In this study, governance refers to “who makes decisions and how decisions are made, from national to local scale, including formal and informal institutions and rules, power relations and practices of decision-making” (Larson and Petkova p.87 2011). Good forest governance is built around principles such as accountability, inclusion and transparency (Kanowski and Cashore 2010). Good governance is also a form of political decision-making that emphasizes legality, legitimacy, and participation of citizens and governments in formulating and implementing policies, such as for REDD+ (Forsyth 2009). Even though REDD+ is not primarily a governance reform, it will affect or be affected by forest governance, it can improve forest governance or be undermined by its failures and, therefore, it depends on good forest governance if it is to be efficient, effective and equitable (Larson and Petkova 2011). Multilevel and multi-actor governance are sometimes referred to normatively, as they are considered, if effective (Larson and Lewis-Mendoza 2012), to be more inclusive, coherent and participatory than top-down governance. They take into account both vertical (multilevel) and horizontal (multi-sectoral, multi-actor) coordination.

The general starting point of a multilevel governance approach is that we witness a series of reconfigurations of the relationships and modes of interactions between central states, other levels of government and other actors. According to some multilevel governance theorists, states are no longer the monopolizing or even necessarily the central actors of policy-making; rather the power of government can increasingly be shaped by and shared between actors operating at multiple levels (Saito-Jensen 2015). As a consequence, the role of the state is being transformed as state actors develop new strategies of coordination, steering and networking (Bache and Flinders 2004). In other cases, civil society can begin to take on the role of the state, especially regarding environmental issues linked to a global economic market (Keck and Sikkink 1998). Gereffi and Mayer (2004) document this co-evolution of market, state and societal institutions in global governance. Some theorists, however, question the dichotomy between “state” and “civil society” (Fox 1996; Evans 1996). Notably, in this research we found important hybrid actors, who moved among institutions—state, civil society and the private sector—to play key roles in REDD+ debates.

REDD+ initiatives are emerging worldwide across vertical and horizontal levels of governance similar to what we have seen with forest certification schemes in the 1990s and early 2000s through the emergence of non-state market-driven governance systems. Through the development of timber certification schemes by international non-governmental organizations and the timber industry, governments worldwide began to participate in the movement to improve forest management to implement environmentally and socially responsible practices (Cashore 2002). In turn, voluntary rules of these certification schemes or soft policy standards, in some cases, have become hard policy or obligatory at a federal or national government level.

This is relevant to REDD+ in Peru, which developed along two parallel trajectories: NGOs and the private sector developed REDD+ projects to reduce deforestation and forest degradation on a voluntary basis and with little interference by the government, while the state attempted to streamline the national REDD+ strategy and its rules for development. This arrangement has given rise to non-state actors becoming more involved in land and forest policy decisions. Cole (2011), Nagendra and Ostrom (2012) and others argue that polycentric solutions, in which the role of the state is equal to those of other actors, are particularly relevant to REDD+. If multilevel governance focuses attention on multiple levels and actors, inside and outside of the state, this situation poses the challenge of specifying new mechanisms of control and accountability among these actors (Saito-Jensen 2015). This is particularly clear in Madre de Dios, due to the distrust of NGOs in the region by the indigenous federations and specific land-user associations. This research suggests that the role of the state in Madre de Dios—and nationally—remains central to what is possible and what is effective. REDD+ cannot be done without the state on board. In Peru, in 2015, San Martin and Madre de Dios were established as the first pilot regions.

The idea of bringing together multiple actors and sectors—in order to build a common vision, for a country or for a landscape—is now also central to REDD+. As Thompson et al. (2011 p.105) state, “the enactment of REDD+ programs in specific places will require the alignment of the viewpoints and needs of many different actors toward a shared goal of limiting climate change and its human impacts.” There is little question of the importance of this aspect. Research at both national (Brockhaus et al. 2014) and subnational levels (Ravikumar et al. 2015a) demonstrates that challenging business-as-usual development requires REDD+ to engage not only with the conservation sector but also the sectors driving deforestation and degradation. Top-down solutions encounter problems, such as resistance among local actors, which impedes progress (Moeliono et al. 2014; Sanders et al. 2017), although even highly centralized spaces can give people room to maneuver and try new things (Pham et al. 2014). Other studies have demonstrated the importance of forest users’ and inter-community forestry associations’ participation in REDD+ activities along with the support of government agencies and higher-level institutional arrangements (Kashwan and Holahan 2014).

Multi-stakeholder collaboration, across sectors and levels, is one of the core concepts of “integrated landscape approaches” (Kusters 2015; Denier et al. 2015; Minang et al. 2015). In spite of the recognition by these authors that power relations may be unequal and that this needs to be addressed, there are other problems with the concept both of “integration” and of “landscape.” Among other things, both terms are somewhat vague and open to multiple interpretations (Mccall 2016). With regard to the former, Mccall (2016) argues that past experiences with integrated landscape approaches failed not because actors failed to understand the importance of coordination and cooperation but rather because of “issues of power and authority and sufficient control over the holistic landscapes.” With regard to the latter, the author argues that the concept of “territory” establishes a formal institutional basis for this power and authority, in a way that “landscape” does not; he specifically emphasizes the power of indigenous people and communities that hold, or should hold, tenure rights. Mccall mentions, but gives much less importance to, the relevance of state administrative jurisdictions in the concept of territory. Both are relevant to this study.

## Landscape and Drivers of Deforestation in Madre de Dios

The region of Madre de Dios is located in the southeast Amazonian section of Peru along the border of Brazil and Bolivia. It is comprised of 8,518,396 hectares and has a population of 124,000 individuals (Diaz 2013). The region is also known to have the country’s highest population growth rate (4.8%) (GCF 2012). This can partially be attributed to the fact that Madre de Dios’ regional neighbors are Puno and Cusco, regions with one of the highest percentage of extreme poverty in the country and also one of the highest percentage of emigration rates (Gordillo 2014).

Currently, the economic activities of great importance to this region are listed as forestry (for timber, Brazil nuts and latex), mining (gold), agriculture, hydrocarbons and energy, and tourism (Sanchez Espinoza 2013). The particular characteristics of Madre de Dios result in a mixture of opportunities and risks for land-use trajectories. Important sustainable economic opportunities exist in ecotourism, Brazil nuts (a particularly high value non-timber forest product), fish farms and timber. It is notable that no <15 REDD+ initiatives were said to be operating in the region at the beginning of this research (MSAR personal communication 2013).^4^Nevertheless, gold mining and related massive immigration are driving rapid changes to the landscape, as occurred historically with latex and agriculture.

Madre de Dios is often referred to as the “capital of biodiversity” and the regional government has invested in fomenting its eco-tourism sector (Sanchez Espinoza 2013). In order to protect the region’s ecosystems, the government has created six national protected areas, which cover 44% or 3,762,942 hectares of the region. Forest concession areas cover an extension of 1,289,844 hectares, which represents 15% of the total area in Madre de Dios and are located in the three provinces of Tahuamanu, Tambopata and Manu. Approximately 4.46% of land is denominated under agriculture use (Sanchez Espinoza 2013) that is largely found alongside the Interoceanic highway. In Madre de Dios there are 33 native communities legally documented in the region, only seven of which still lack property titles (DRA-GOREMAD 2017, cited in Cruz et al. 2017). Their total area is comprised of approximately five percent of the region.

Deforestation is on the rise in Madre de Dios. Official data from the Ministry of Environment (MINAM 2016) shows that the region lost an average of 5% of forest per year from 2000 to 2014, with the area lost in the second half of that period almost double that lost in the first half (82,118 hectares and 45,600 hectares, respectively). Almost 16,000 hectares of forest were lost in 2014 alone in the region, the highest in the past 15 years (MINAM 2016). According to the 93 interviews conducted in our study, the major drivers of deforestation were: (1) the expansion and recent paving of the Interoceanic Highway, (2) uncontrolled immigration and urbanization, (3) gold mining, (4) land invasions from migrant miners and farmers, and to a lesser extent (5) fires from slash-and-burn agriculture.^5^A distinguishing factor regarding mining and agriculture is that agricultural practices are considered a slow driver of deforestation while current mining practices in Madre de Dios, which are also tied to uncontrolled migration and land invasions, are considered to be driving deforestation at a faster rate (Rodriguez-Ward 2014).

The main underlying deforestation drivers associated with land invasions of illegal loggers, miners and farmers were exacerbated in 2005 due to the expansion of the Interoceanic highway and its subsequent paving in 2010, and the continuous rise in gold prices in the international market from 2008 (Rodriguez-Ward 2014). This increased migration occurred simultaneously with the decentralization process in the region. Many interviewees, including government officials, felt the regional government was ill-prepared and unable to control the influx of migrants (ibid). It was nearly impossible for local and regional governments to hold illegal land users accountable for deforestation, contamination and unsustainable management practices while their offices underwent a restructuring process and lacked support from the central government. An additional underlying deforestation driver is insecure land tenure and overlapping land titles or concessions—problems created in part while these responsibilities were still in the hands of central government, then handed down under decentralization. In 2013, there were over a million hectares or about 20% of the region suffering from some kind of overlap (Sanchez Espinoza 2013). Recent research showed that such overlapping also occurs because different national institutions with weak inter-sectorial coordination grant incompatible land use rights in the same area (Gordillo 2014).

## Methodology

This article stems from primary and secondary data collected for the Global Comparative Study of Multi-Level Governance and REDD+, Carbon Management and Land Use Decisions conducted by the Center for International Forestry Research (CIFOR) within the Madre de Dios site in Peru. The goal of the study was to understand how decisions affecting forests, land use, and benefit sharing are made, who influences whom, how ideas change and whether or not REDD+, REDD+ discourse or REDD+ proponents are having any impact. This study was conducted in three regions of Peru (Madre de Dios, Ucayali, and San Martin). Fieldwork was conducted in Madre de Dios from July 6, 2013 to October 18, 2013 and involved interviewing various multilevel actors from nine of the eleven districts of Madre de Dios and in Lima.^6^For this study we completed 93 interviews with key actors and used four types of field interviews: (1) key informant invterview at the regional level, (2) key informant interview at the district level, (3) in-depth interviews of a specific land-use practice, and (4) interview on benefit-sharing structures and processes. Key informant interviews were conducted first with individuals from NGOs, government, and research agencies and were used as a site selection instrument in order to identify sites of increasing and decreasing carbon emissions; understand the general context of deforestation and REDD+ initiatives in the region; and document actor interactions regarding land use and REDD+. There were 25 key informant interviews conducted at the regional level and seven at the district level with local government officials. Potential interviewees had been identified through a scoping study of the region, and the remaining key informants were selected through snowball sampling. There were 47 in-depth interviews conducted that focused on actors directly involved in land-use changes. These studies were used to collect data on the detailed history of the land-use change at the local level and actor interactions regarding land-use change and REDD. The remaining 14 benefit-sharing interviews applied only to actors directly involved in benefit sharing arrangements in REDD+ initiatives. Key informant interviews and in-depth interviews averaged 1.5 h while benefit-sharing interviews were longer in length, averaged 3 h, and usually had to be conducted in two visits. Questions were semi-structured and open-ended. Additional data for this study was gathered through participant observation by attending various multilevel actor meetings and presentations held within Madre de Dios during the timeframe. Interviews were recorded with the informants’ permission, fully transcribed and coded using the qualitative analysis software of NVivo into a database.

### Informants Interviewed

In this study 93 interviews were conducted in 46 different institutions. The institution types represented (from highest to lowest number of interviews) are: 15 government institutions (35 interviews), 9 NGOs (21), 7 land-user associations (11), 4 concessions (6), 3 communities (8), 3 private companies (5), 3 research institutes (4), one indigenous association (1), and one humanitarian organization (1). Multiple interviews were conducted with some institutions in order to gain different perceptions of land-use changes in the region. For example, there were a number of regional government and NGO employees who also served on the REDD+ Working Group committee or were directly involved in a REDD+ initiative. Different types of interviews (i.e. key informant, in-depth, and benefit-sharing) were also conducted in one institution once key actors and REDD+ projects were identified. Table 1 demonstrates the level (local, regional, national, and international) and types of institutions interviewed as well as the number of interviews conducted. We interviewed 34 individuals representing national agencies, 29 individuals from local agencies, 25 individuals from regional agencies, and 5 from international agencies. There are two NGOs that we have categorized as belonging to the international/ national levels as they are international organizations with national offices in Peru. We also found that some interviewees belonged to more than one category (i.e. a concessionaire and member of a land-user association or a National Service of Natural Protected Areas (SERNANP) park guard and a community member^7^

**Table 1:** Institution and actor interviews

No.	Scale	Type	Institution Name	Interviews
1	Local	Community	Arca Pacahuara community	3
2	Local	Community	Comunidad Nativa Infierno	4
3	Local	Community	Sandoval Settlement	1
4	Local	Concessionaire	Forest concession	1
5	Local	Concessionaire	Sonidos de la Amazonia Eco-turismo concession	1
6	Local	Concessionaire	Tropical Woods concession	1
7	Local	Concessionaire	Brazil nut concession	3
8	Local	Government	Gobierno Distrital de Iberia	1
9	Local	Government	Gobierno Municipal Inambari	2
10	Local	Government	Gobierno Municipal Tambopata	2
11	Local	Government	Gobierno Provincial Tambopata	1
12	Local	Government	Gobierno Provinicial Tahuamanu	1
13	Local	Land user Association	Alegria Brazil Nut Association	1
14	Local	Land user Association	Tambopata Environmental Management Committee	1
15	Local	Land user Association	Asociación de Mineros Artesanales de Taurfaticos (AMATAF)	1
16	Local	Land user Association	Asociación de productores agrarias y lavadores artesanales de oro del Rio Malinowski (APAYLOM)	2
17	Local	NGO	Arbio	1
18	Local	NGO	Huaruyo	1
19	Local	Private company	Tambopata Tours	1
20	Regional	Government	Dirección Regional de Agricultura (DRA)	2
21	Regional	Government	Dirección Regional de Energía, Minería e Hidrocarbonos (DREMH)	2
22	Regional	Government	Dirección Regional de Fauna y Flora Silvestre (DRFFS)	1
23	Regional	Government	Gerencia Regional de Recursos Naturales y del Medio Ambiente (GRRNYMA)	7
24	Regional	Government	Gobierno Regional de Madre de Dios (GOREMAD)	2
25	Regional	Humanitarian	Fundacion Caritas	1
26	Regional	Indigenous Association	Federación Nativa de Madre de Dios (FENAMAD)	2
27	Regional	Land user Association	Federación de Agricultores de Madre de Dios (FEDAMAD)	2
28	Regional	Land user Association	Federación de Forestales de Madre de Dios	2
29	Regional	Land user Association	Federación de Productores de Castaña de Madre de Dios (FEPROCAMD)	2
30	Regional	Private company	Conservacion Ambiental y Desarrollo en el Peru (CAMD)	1
31	Regional	Research	Universidad Nacional Amazonica de Madre de Dios (UNAMAD)	1
32	National	Government	Ministerio Nacional del Ambiente (MINAG)	2
33	National	Government	Organismo de Supervisión de los Recursos Forestales y de Fauna Silvestre (OSINFOR)	1
34	National	Government	Proyecto Especial Madre de Dios (PEMD)	4
35	National	Government	Servicio Nacional de Áreas Naturales Protegidas (SERNANP)	5
36	National	NGO	Asociación para la Conservación de la Cuenca Amazónica (ACCA)	8
37	National	NGO	Asociación para la Investigación y el Desarrollo Integral (AIDER)	4
38	National	NGO	Peru Bosques	1
39	National	NGO	Sociedad Peruana de Derecho Ambiental (SPDA)	1
40	National/ Intl	NGO	Rainforest Alliance	1
41	National/ Intl	NGO	World Wildlife Fund (WWF)	3
42	National	Private company	Bosques Amazónicos SAC (BAM)	3
43	National	Research	Instituto de Investigaciones de la Amazonia Peruana (IIAP)	1
44	International	Government	German Agency for Technical Cooperation (GIZ)	2
45	International	NGO	Frankfurt Zoological Society	1
46	International	Research	Consorcio Madre de Dios	2
				93

### Field Sites

**Fig. 1:**
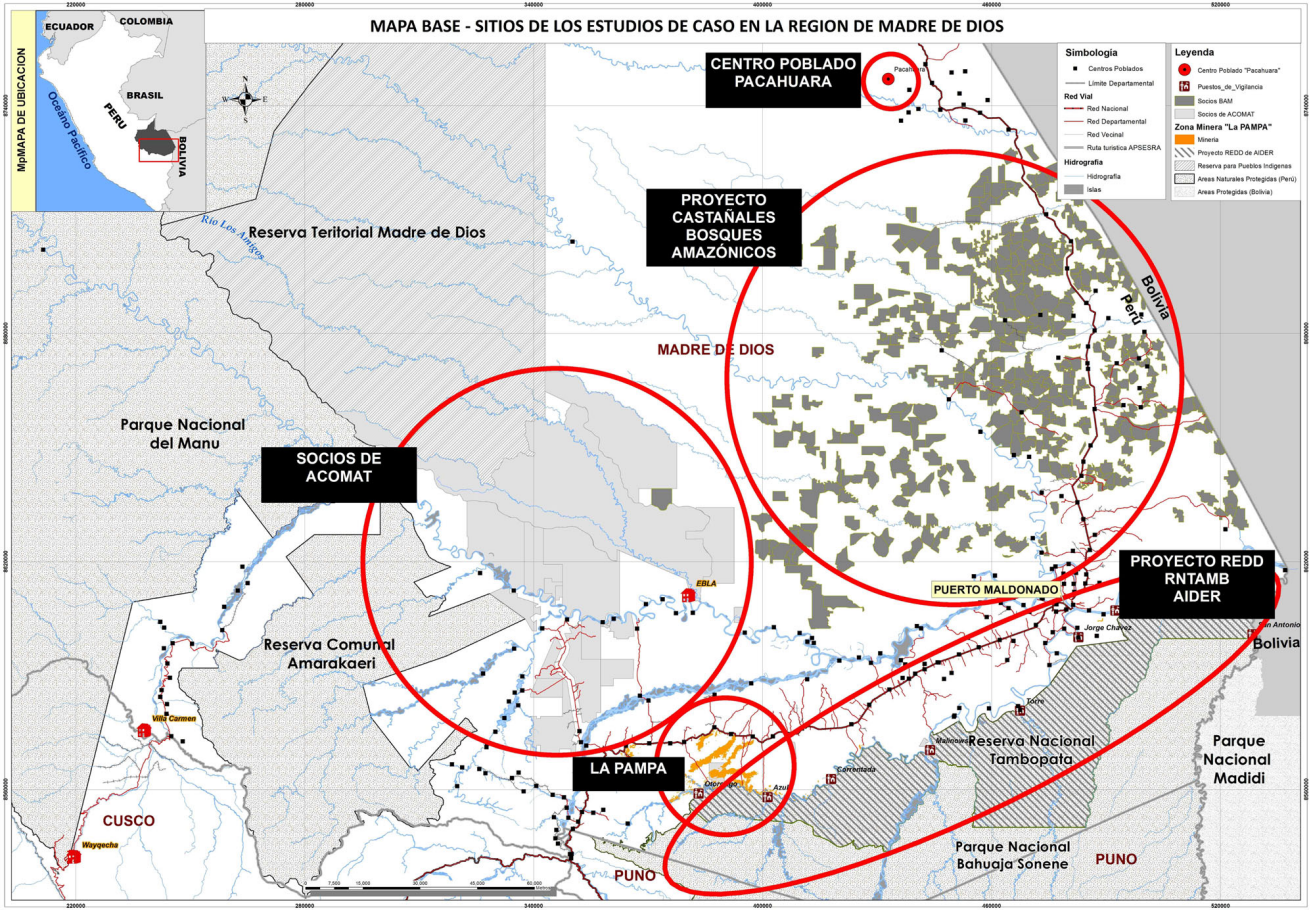
A map location of five case studies for increasing and decreasing carbon emissions sites

Of the 93 interviews, 61 focused specifically on the five case study sites. The sites were selected following the study’s field research methods training guide (Ravikumar et al. 2015b) and based on data provided by key informant interviews; maps demonstrating a land-use change resulting in sites with high and low carbon emissions; inclusion of an affected rural population; a site affected by a REDD+ project and benefit sharing arrangements; and the feasibility of conducting interviews and site visits within the timeframe. The five sites selected include three “decreasing carbon emission sites” (two REDD+ initiatives with benefit sharing arrangements and one REDD+ initiative in the process of becoming certified) and two classified as “increasing carbon emissions sites” where deforestation and forest degradation were occurring without efforts to curtail this. See Fig. 1 and Table 2.

**Table 2:** The five case studies for increasing and decreasing carbon emissions sites

Name	Emission type	Actor type	Land type (Hectares)	Time span
AIDER-SERNANP REDD+ Project	Decreasing	NGO, Governmental agency	National Protected areas (573,299 has)	2008–2028
BAM- FEPROCAMD REDD+ Project	Decreasing	Private company, Land user association, and concessionaires	Brazil nut concessions (291,566 has)	2009–2040
ACOMAT initiative	Decreasing	NGO and individual concession owners	Ecotourism, conservation, timber and NTFP concessions (316,282 has)	Initiated in 2012
La Pampa Mining zone	Increasing	Illegal and informal gold miners	Tambopata National reserve buffer zone and surrounding area (~ 2000–5000 has)	Peaked in 2008
Arca-Pacahuara Agriculture	Increasing	Religious farming community	Forest conversion to farmland (3500 has)	1995

*ACOMAT* Association for timber and non-timber Concession holders in the provinces of Manu and Tambopata, *REDD+* Reducing Emissions from Deforestation and Degradation plus carbon sequestration and promoting sustainable forest management. All remaining acronyms are listed in Table 1.

The first decreasing emission site is the AIDER-SERNANP REDD+ project. It includes the national Association for Research and Integrated Development (AIDER; an NGO) and the National Service of Natural Protected Areas (SERNANP; a governmental agency) as co-administrators of two national protected areas (Tambopata National Reserve and Bahuaja Sonene National Reserve) from 2008 to 2028. The project is Verified Carbon Standard and Climate, Community and Biodiversity Alliance (CCBA) verified and is located in the province of Tambopata. It encompasses a total of 573,299 hectares of primary forest. In 2013, AIDER secured payments for carbon credits with Pacifica Seguros (a Peruvian insurance company) to be invested in monitoring, research and community outreach projects for the protected areas and settlements located within the park’s buffer zone (including the settlements of Nueva America and Sandoval, and the native communities of Palma Real and Sonene).

The second REDD+ project includes the partnership of Bosques Amazónicos (BAM; a private, national company), the Regional Federation of Brazil nut collectors (FEPROCAMD), and 377 Brazil nut concessionaires. The BAM-FEPROCAMD REDD+ project is located in the Provinces of Tahuamanu and Tambopata, encompasses a total of 291,566 hectares of primary and secondary forest and spans for a period of 31 years (until December 31, 2040). The contracts between participants were set up so that payment for carbon credits will be made directly to BAM, which will disperse 30% of the income to individual concessionaires, while they retain 70% of the carbon payment profits. Additional benefits in the contracts signed between Brazil nut concessionaires and the Federation will disperse 70% of profits from a Brazil nut-processing plant to be constructed by BAM and with the latter receiving the remaining 30% of profits.

The third case study includes the Association for timber and non-timber concession holders in the provinces of Manu and Tambopata (ACOMAT), which is comprised of eleven concessionaires which consist of four different land-use types (eco-tourism, conservation, timber and NTFP). It encompasses an area of 316,282 hectares in the two provinces of Manu and Tambopata. ACOMAT is spearheaded by the Association for the Conservation of the Amazon Basin (ACCA; a national NGO). This case study was chosen as a site of decreasing carbon emissions as the NGO plans to deter deforestation and degradation caused by land invasions of illegal miners and recurrent incursions of illegal loggers in their areas while improving their current land use through sustainable forest management techniques (for example, implement FSC certification, agroforestry and aquaculture alternatives, payment for environmental services, and increase conservation areas and/or reforestation with native species within concessions).

La Pampa is the first case study of increasing carbon emissions due to artisanal and small-scale gold mining activities. “La Pampa” is an informal denomination of an area greatly affected by illegal and informal mining. It is situated approximately between kilometers 80 and 140 of the Interoceanic Highway (located within the districts of Laberinto and Inambari, in the Tambopata province). Those who are mining in La Pampa do so within informal mining areas as well as in mining, reforestation, ecotourism and conservation concessions and farmland, which are located within the Tambopata National Reserve buffer zone (all of the area in the southern margin of the highway) (Gordillo 2014). There is no consensus as to how many hectares, concessions and operations are currently underway in La Pampa as much of the mining is informal, illegal, furtive and conducted mostly by temporary migrants, also referred to by locals as “floating populations of migrant miners.” Some estimate that there are close to 10,000–15,000 miners working in La Pampa in 2013 (Rodriguez-Ward 2014). While one source estimates that by 2013 there were 5,000 hectares affected by mining activities solely within the Tambopata buffer zone (anonymous personal communication 2013) another estimates that between 2004 and 2011 ~2297 hectares were converted into mining areas within the Tambopata buffer zone, mostly outside concession areas and around access routes such as the Interoceanic Highway and major rivers. Surprisingly there are also important forest regrowth processes taking place due to the ecosystem resilience and mostly in abandoned farming areas (Gordillo 2014).

The second case study of increasing carbon emissions is the religious farming community of Arca Pacahuara. To date, the community, whose members originate from Puno and Cuzco, has converted 3500 hectares of forest for the cash crop of corn through substantial financial and technical assistance from regional and national governmental agents. The community totals 2500 inhabitants and was formed in 1995 in the district of Iberia. At the time of the research, community leaders had petitioned the Regional Agriculture Department to extend their area by 26,000 hectares in hopes to increase their farming potential. Unlike La Pampa, Arca Pacahuara is located on legally titled land (although land disputes do exist with two of their forestry concession neighbors), and the community has received financial and technical support from regional and national governmental entities for their production activities. The mining problem in La Pampa is far more complex. However, the mining sector in general has been historically considered a central pillar of the Peruvian economy (MINAM 2009).

## Results

We discuss the results in four sections. First, we discuss how the REDD+ initiatives and working groups were implemented in the region. Second, we examine which important actors were included and excluded from REDD+ initiatives in the region. Third, we address which challenges REDD+ and its actors have encountered in Madre de Dios. Fourth, we describe the coalitions that emerged due to these actors’ relationships and REDD+ initiatives in the region.

### REDD+ Initiatives and Working Groups

According to interviewees, national and local civil society members along with regional governmental officials have played an important part in the implementation of REDD+ in Madre de Dios. In 2008 the Regional Government of Madre de Dios, along with research institutions and private businesses, created the inter-institutional group called “Consorcio REDD.” Their main objective was to establish the historic deforestation rate of Madre de Dios in order to facilitate baseline data for REDD initiatives. After two years, the group dissipated due to a lull in funding. By the beginning of 2011, the impetus to begin REDD+ activities re-emerged with an initiative led by the Regional Government and civil society actors working hand in hand. The group was reactivated under the name of REDD+ Working Group with the goal to provide regional baseline data and reference levels for the advancement of REDD+ initiatives and to increase local knowledge on REDD+ (i.e., information dissemination and capacity-building). In 2013 the REDD+ Working Group was legally recognized through a regional decree (Ordenanza Regional No 015-2013-RMDD-CR) thus giving it more legal weight and decision-making power.

In 2012, the Working Group created a two-year operational plan in which they defined their organizational structure and created commissions and sub-commissions that assigned members with specialized backgrounds in REDD+-related themes and defined specific tasks. As of 2012, it was composed of a President (the Director of the Regional Office of Natural Resources and Environmental Management, (GRRNYMA), a Communications Secretary (AIDER-NGO), and a Technical Secretary (World Wildlife Fund-NGO). There were four commissions that were headed by multiple actors: the Safeguard commission (Sustainable Development Department-Regional Government of Madre de Dios), the Deforestation Baseline commission (Ministry of Environment- national government), the Work plan commission (Peruvian Society for Environmental Law-NGO), and the newly created Regulation commission (the leading organization was still undetermined at the time of the study). The Deforestation Baseline Commission also oversaw the four sub-commissions of Biomass calculations and carbon mapping (WWF), Deforestation analysis (AIDER), Deforestation modeling (AIDER), and Deforestation Drivers’ analysis (Ministry of Environment). Aside from GRRNYMA, the Working Group members participated voluntarily and were not financially remunerated.

At the time of the research, the Working Group had several key accomplishments. In 2011, it strengthened local capacity training on REDD+ through a certificate course on monitoring, reporting and verification (MRV) offered in conjunction with the University of Madre de Dios. This free course was offered to regional government employees, regional committee council members, as well as to relevant civil society actors. In 2012, various Working Group members and those already involved in REDD+ projects collected field data for biomass and carbon analysis in 608 plots throughout Madre de Dios. This data was used to measure carbon in different vegetation types and gather information for a regional carbon map finished in 2013.

Another equally important but separate REDD+ working group in Madre de Dios was the Indigenous REDD+ Working Group. It was created under the direction of the Interethnic Association for the Development of the Peruvian Rainforest (AIDESEP; national indigenous organization) and the Native Federation of the Madre de Dios River and its Tributaries (FENAMAD; a regional indigenous organization), with financing from the International Development Bank to focus on promoting the inclusion of indigenous communities in REDD+ projects. There was a simultaneous creation of indigenous REDD+ working groups in various regions of Peru by AIDESEP and the Coordinator of Indigenous Organizations of the Amazon River Basin (COICA) (Che Piu and Menton 2013). Originally the Indigenous REDD+ Working Group’s stance was to oppose the commercialization of carbon and payments for ecosystem services as no law existed to guide how funding from carbon payments would be made, and out of fear that other actors, particularly NGOs and the government, would redirect the benefits toward themselves. Negative experiences with external actors, such as the case of the “carbon cowboy” in Loreto,^8^also contributed to the aforementioned fear and mistrust (REDD Monitor 2012). A FENAMAD representative stated, “it was better to funnel REDD+ financing directly to indigenous federations instead of other actors, as they have indigenous communities’ best interests at hand” (anonymous personal communication 2013). The Indigenous REDD+ working group was initially interested in using funds to resolve pending land titling in the region, which is a necessary requirement for REDD+ activities, but was also a salient issue for several native communities. Funding was later used to create projects with the goal of incorporating carbon credits. There was very little interaction observed between the two REDD+ working groups during the time of fieldwork, and conflicts arose between REDD+ projects within indigenous communities that did not invite FENAMAD to participate.

### Actors and Their Role in Shaping REDD+

In this section we examine actors identified by the 93 interviewees as important REDD+ actors in the region; why they have chosen to participate in REDD+ activities; and how their role affects the decision-making process and the direction of REDD+. We found that REDD+ initiatives in Madre de Dios were initiated through the combined efforts and partnerships of actors from multiple levels and sectors, comprised of private companies, concession owners, indigenous communities, regional, national and international NGOs, Indigenous federations, land-user groups and associations, national governmental agencies, and international donors, but at varying levels of participation and decision-making power. The majority of interviewees in the region identified the private companies and NGOs as the main champions for REDD+, with the regional government gradually increasing its participation and influencing the shaping of the REDD+ process. While certain land-user groups, indigenous community members, and district level government officials were identified as having less participation in the REDD+ Working Group, REDD+ was seen as bringing together multi-scalar and multi-sectorial actors that under normal circumstances would not interact with one another. The dialog created with the different stakeholders also brought the discussion of potential solutions to current and future land-use change leading to deforestation in critical areas such as La Pampa mining zone (Gordillo 2014).

These interactions also had potential to propagate certain inter-regional alliances and international relationships with incentives, benefits and burdens varying among actors. Notably, not every actor can be easily classified as “state”, “private sector” or “civil society”. Rather, certain individuals can be categorized as multi-level hybrid actors, as they moved from one category and/or level to another. This blurs the definition of who is shaping landscape governance in the region, while suggesting that lessons and understanding from one “location” or perspective might be carried on to another. We will come back to this in the discussion.

### Civil Society

Early on, environmental NGOs were the main promoters of REDD+ initiatives in the region. During the time of the study, the Peruvian branch of WWF was the Technical Secretary for the Environmental Services and REDD+ Roundtable (Spanish acronym: MSAR) and was repeatedly identified by the interviewees as the main entity promoting REDD+ in the region. Although the Director of the Regional Governmental office of the Environment (GRRNYMA) held the most important position as President of the REDD+ Working Group, he depended heavily on guidance and technical assistance from WWF. This was partly due to the lack of support from the national Ministry of Environment, and partly due to the ongoing loss of institutional memory, as regional government employees constantly move between positions and offices. WWF has also taken a leadership role in REDD+ by organizing capacity training for governmental and nongovernmental employees. AIDER and ACCA, two NGOs, are also important participants as they provide technical assistance in fieldwork and mapping activities and also have their own REDD+ initiatives. NGOs have also directed financial and technical resources to the regional government of Madre de Dios, in order to complete Working Group activities. In 2012, WWF financed the installation of vegetation type plots for the Working Group biomass study and in the same year, AIDER completed a capacity-training workshop for Regional Government personnel in the Working Group’s Biomass sub-commission.

### Private Sector

Personnel from the private company Bosques Amazónicos (BAM) office in Puerto Maldonado were not seen as vocal or visible actors in the MSAR as the company faced financial issues in 2011 and again in 2014 leading to a restructuring of positions and inability to dedicate time to the Working Group meetings. However, staff from BAMs’ Lima office played an integral part in promoting REDD+ at regional and national level, as they were active participants with the national and regional REDD working groups; created strong ties with Verified Carbon Standard representatives; and collaborated with the Governors’ Climate and Forest (GCF) Task Force. While BAM is a private company, it was also seen by some stakeholders as a socially and environmentally responsible company, interested in sustainable management and investing in community development, rather than a traditional for-profit entity. However, other actors had a different opinion, based on how they negotiated contracts with the Brazil nut concession owners, where they were seen as trying to maximize their corporate profit. The timber companies MADERACRE and MADERIJA had an active REDD+ project and had advanced faster than other projects, possibly because they had fewer decision-makers in their chain of authority. However, they were not identified as active Working Group participants and their focus was more on individual business interests.

### Government

While national and international NGOs play an important role in the development and financing of REDD+ activities as stated by interviewees in the region, the Regional Government Department of Natural Resources and the Environment (GRRNYMA) was identified as an new important actor in REDD+. GRRNYMA presided over the REDD+ Working Group, oversaw its activities, and held many of its meetings at their office. The regional government was also responsible for passing the ordinance that established the Working Group. Additionally, the regional representative of the national office of the Ministry of Environment (MINAM), although solely focused on mining issues, attended MSAR meetings and was extremely knowledgeable about REDD+ activities and reducing carbon emission initiatives. This official shared office space with GIZ (a German international development organization), and both focused on low impact mining activities. While this official had frequent interaction with the regional government office of mining (DREHM), Proyecto Minero, and mining unions, there were no DREHM representatives at the REDD+ meetings.^9^Nor were the regional government officials in charge of agriculture (DRA) involved in the MSAR.

### International Donors

International donors—the Gordon and Betty Moore Foundation, U.S. Agency for International Development, Norwegian Agency for Development Cooperation (Norad) and KfW development bank—were noted as important actors in funding national and regional agencies, which then re-invested and channeled a portion of the funds to regional and local REDD+ actors and their activities. This financing assisted in the start-up costs for REDD+ planning, baseline studies, and initiative implementation as few projects actually received payments for carbon. The Moore Foundation and KfW were important donors to the national office of the Ministry of Environment and its National Program for Forest Conservation and Climate Change Mitigation. Funds from this program were invested in hiring technical experts in the regional environmental government office, who participated in the Working Group activities. In 2010, WWF used Norad funds to reactivate the Working Group in Madre de Dios and to conduct capacity-building workshops for local actors. USAID provided funds to the agency Peru Bosques, which supported the creation of the new Regional Environmental Authority (ARA) office.

### Multilevel Hybrids

Not all actors fit neatly into the categories above. Rather, we found a high incidence of “job jumping”—a kind of revolving door between these actors in governmental, NGO and private sector positions—partly associated with institutional instability. We define these individuals as “multilevel hybrids”. While theory states that actors influence the decision-making process based upon the interests of their workplace or actor type (McDermott et al. 2012; Brockhaus et al. 2014), there were various individuals who pushed the decision-making process due to individual interests and ideals as well, from varied workplaces and actor “types”. In our research, we found five individuals who could be defined as multilevel hybrids. For example, an ex-BAM employee (2013) who became a representative of the Ministry of Environment (2014) was highly influential in promoting REDD+ in Peru, connecting international actors (i.e., Verified Carbon Standards certifiers, Global Climate Facility representatives, and Brazilian government officials) to regional actors (GRRNYMA and MSAR). In addition, in Madre de Dios there were highly idealistic young environmentalists in various types of positions rotating between different institution types (i.e., WWF, AIDER, GRRNYMA). While there was only a small number of individuals we would consider multilevel hybrids, the small number does not negate their importance.

Also, it should be noted that there were various NGOs that could be categorized as international, national and regional. The national NGO AIDER had its headquarters in Lima but also regional offices in Madre de Dios and Ucayali. The international NGO WWF had a national office in Lima and a regional office in Madre de Dios. The NGO ACCA had an office in Washington DC and several regional offices in Peru. Thus, we found that interests and funding originated from multi-scalar actors, suggesting more fluidity or gray area than fixed categories might suggest. Decision-making trickled up and down depending on priorities and needs.

### Missing and/or Excluded Actors

The first actors noted missing from the Regional REDD+ Working Group were individual land users and their associations, specifically miners, Brazil nut producers, and community members living in the buffer zones of national parks. The regional federation of Brazil nut collectors (FEPROCAMD) was a partner with BAM in its Brazil nut REDD+ project and represents the concessionaires, but was not identified as a main REDD+ actor due to limited participation in the REDD+ Working Group meetings. Their office reported that their few employees were already spread thin working with over 300 concessionaires in various locations. The Tambopata Environmental Management Committee played a role in AIDER’s REDD+ project, but was not identified as a main actor in REDD+. The committee consisted of land users who were not decision-makers on the Project Design Document, although they reviewed the project’s activities and budget at annual meetings along with an important partner, the National Service of Natural Protected Areas (SERNANP). This checks-and-balance scheme was already set up before the REDD+ project. The mining associations, AMATAF and APAYLOM, were also located within AIDER and SERNANP’s REDD+ project buffer zone. Representatives of both associations complained of little interaction with AIDER even though they were included in their Project Design Document. Miners in general are important actors in the region as they are regularly labeled as main actors in deforestation and degradation. However, AMATAF and APAYLOM were significantly different than most other gold miners; they were in fact historical dwellers of the region who had practiced artisanal mining for decades with a lower impact than the current gold miners. In addition, these stakeholders sometimes acted as an active barrier to prevent further incursions from wildcat gold miners into the natural reserve.

With regard to other actors left out of REDD+ processes, neither farmer associations nor indigenous associations were represented in the MSAR. The farmer associations were excluded because there were no opportunities for the agricultural sector in REDD+ projects. Furthermore, their leader had personal issues with the justice system, which forced him to maintain a low profile. Indigenous associations were not represented due to their own choice. One person sometimes attended the meetings on behalf of FENAMAD, but he did not have any decision-making authority on behalf of the federation. As mentioned previously, there was little interaction between the MSAR and the indigenous working group, which was generally seen as a problem.

Other local actors that were notably absent from the REDD+ Working Group were local government officials (i.e., provincial, municipal, district). The Municipal government’s Environmental Management office located in the capital city of Puerto Maldonado complained that they were not invited to the meetings. The mayors of Masuko and Iberia, where the majority of mining and forest concessions are located, knew very little about the existing REDD+ projects within their jurisdictions. The only district level government official that had some knowledge of REDD+ was the mayor in Inapari, who was in regular communication with officials of the Proyecto Especial de Madre de Dios (PEMD). PEMD actively participated in MSAR and ARA discussions and had offices in the city of Puerto Maldonado (where Working Group meetings were held) and an office in Iberia (close to the mayor’s office). PEMD is also a well-connected actor to multiple levels of government and civil actors due to their multilevel composition and funding. Regional government officials in the REDD+ Working Group said that local government officials were invited, but that they never attended due to the lack of funds or a lack of interest, as their job responsibilities do not include REDD+ activities. Hence attendance would be self-motivated and non-remunerated.

### Challenges in REDD+ in Madre de Dios

There were multiple challenges in the implementation and promotion of REDD+ and sustainable landscape approaches. These challenges stem, first, from the instability caused by the impacts of decentralization that affected the regional government offices and, second, the struggle to balance power between multiple actors and interested parties. Below we discuss the main challenges cited by the 93 interviewees of the study.

Forestry decentralization is a recent and ongoing process in Peru. As of 2011, eight regional governments of Peru, including Madre de Dios were undergoing forestry decentralization (Weiland Fernandini and Sousa 2015; Che Piu and Menton 2013). Article 51 of the Organic Law of Regional Governments (Ley Organica de Gobiernos Regionales, #27867) transferred control over local forests and land use, including decisions about forest concessions, from the National Ministry of Agriculture (MINAG) in Lima to regional governments.^10^Decentralization suggests that regional governments hold important relevant powers. However, according to the 31 individuals interviewed as key informants, there have been numerous challenges. While decentralization began in Madre de Dios in 2008, most restructuring began in 2010, and by 2013 government workers and the public were still experiencing problems adapting to changes. Regional-level institutions changed their governance structure; functions and responsibilities were transferred from Lima and re-assigned to different agencies. The general sentiment was positive, because decentralization expedited permits (people no longer had to go to Lima); increased efficiency; and created a region-based government that was more directly involved with land users and could, theoretically, better understand their needs. More local level governments, such as provincial and district-level,for their part, felt their daily functions and budgets were not contemplated in nor affected by decentralization; their daily operations had little interaction with national or regional governments.

The regional government, however, was regularly described as having weak sanctioning power due to budgetary restrictions—that is, they had increased authority due to decentralization but insufficient budgets to fulfill their new mandates. Budget constraints led to the “revolving door” of personnel, loss of “institutional memory” in various departments, the inability to train government employees to improve efficiency and response time for land users, and led to a high incidence of corruption among government agents responsible for concession permits. The regional government also inherited problems from the central government—for example, overlapping land titles and permits are rampant in Madre de Dios, but there is no single cadaster, and older permits do not have GPS coordinates. Ultimately these problems affected the control and regulation capacities of the regional government.

The transition in adopting decentralization changes and the redefinition of roles and responsibilities was a time-consuming process. A second attempt at restructuring involved the creation of the Regional Environmental Authority (ARA) in 2013 and led regional governmental employees to be concerned about their job security once again. Land-user associations feared that the central government was allowing NGOs to shape ARA’s structure instead of land users themselves. They protested that the creation of the ARA was done “behind closed doors” and sent letters to the Ministry of Environment office in Lima.

The challenges seen with decentralization and the creation of the ARA parallel the challenges undergone by REDD+ initiatives; the former were underlying the latter. A weak governance structure during this time also led to a fragmentation of the Regional Government of Madre de Dios offices and the inability to solve land conflict disputes, especially land invasions and the overlapping of concession rights. This fragmentation and lack of communication between regional and national, such as in the case of the two regional government offices of mining and territorial planning, and the national office of the Ministry of Environment, also led to the inability to solve the problem of different land measurement tools. This was an impediment to REDD+ implementation, as land tenure security is a requirement for REDD+ initiatives in order to be certified.

Another similar challenge confronted by regional REDD+ initiatives and the Working Group members is related to mistrust between actors, as well as territorial concerns of indigenous communities. Concessionaires and local land-user associations were extremely wary of NGOs’ motivations regarding REDD+ and their perceived increasing power to influence government decision-making and policies. People interviewed were concerned that NGOs were only involved in order to be intermediaries for carbon payments and were channeling funds to this end. This mistrust can be associated with missing accountabilities: NGOs can be important advocates for local populations, but they have not been elected to represent local actors and they cannot be voted out of office. In the case of REDD+ specifically, NGOs have clear interests of their own that do not necessarily coincide with those of current land users.

Specific challenges the REDD+ Working Group members encountered were limited information-sharing by individuals within the REDD+ projects, which delayed decision-making and planning. Paradoxically, while the goal of these projects is a public benefit (to decrease emissions and improve forest management), their funding is based on a market mechanism. Thus, the business aspect of payment for carbon credits made project proponents very cautious about sharing their data amongst each other. Another important hurdle the working group had to overcome was the lack of participation of specific actors involved in, or with authority over, land management decision-making, especially when it came time to vote or assume tasks and responsibilities in the Working Group. Furthermore, during the time of this study, there was no law regulating payments for environmental services (PES) in Peru.^11^

It is notable that by 2013 only three out of the 15 REDD+ projects had advanced to the stage of getting their project design document (PDD) certified and to be able to sell carbon credits. Two more projects were in the design process of their PDD; one initiative was under redevelopment; and nine projects were temporarily halted or had been terminated. Challenges mentioned specifically for sustaining these REDD+ projects included land invasions, a lack of funding, conflicts with proponents and local actors, the inability to demonstrate sufficient deforestation threat to Verified Carbon Standard certifiers, and/or inability to compete with other land uses in terms of profitability, such as gold mining and illegal logging.^12^

### Coalitions for Change

The increased interaction and communication between multiple actors from multiple scales and/or interests was repeatedly brought up by interviewees as an important impact of REDD+ and the REDD+ Working Group. REDD+ was touted as creating a space for dialog between actors who did not traditionally speak to one another before and as being able to foster new relationships and alliances. Interviewees asserted that involvement in REDD+ initiatives and working groups created a space for dialog between different types of actors. One central level government representative stated:

“REDD has brought different actors together who normally don’t communicate with one another. It has brought MINAM closer to people in the regional REDD+ Working Group. The regional and national governments had a gap between them and REDD helped to fill this gap” (Peru Bosques, 2013).

A regional government official reasserted this by stating:

“[Now] we are working with public and private institutions and (we) do not have problems with them. Before we had some problems with NGOs… but the regional government has had an attitude change due to the decentralization process and since then (past five years) we have started to work more in alliance with NGOs. Before you would never see us in the same room or in a meeting with an NGO. This is also partly due to the surgence of REDD projects and the MSAR… REDD has also helped create a space for us (regional government) to work with other regional actors. Before each one of us worked on our own (*cada uno trabajo por su lado*)” (DRFFS, 2013).

We found that participation in the REDD+ Working Group resulted in a type of “regional alliance” that led to a divide between its participants (especially the regional government and non-governmental actors) against the national governmental actors from the Ministry of Environment. This was evident during the creation of the national deforestation maps and reference level model. During the time of the study, the Ministry of Environment was attempting to create a national deforestation map, considered by several stakeholders at the national level as a non-participatory and non-inclusive process (Kowler and Larson 2016). Madre de Dios, however, had already advanced significantly with its own maps ahead of the national government, which in the end chose to use a different methodology. The maps developed by the region represented an interesting example of participatory processes. Many Working Group members had spent considerable time and resources on creating the deforestation and carbon maps, and were having difficulty accepting the Ministry of Environment’s decision.^13^As one representative of the regional government said:

“When the Ministry of Environment told us there was going to be just one way to measure carbon, it was like a nail to the heart because of all the hard work and effort we had put in” (ACCA communication, 2013).”

Another interviewee describes the poor relationship between the national and regional government offices due to this conflict, but stressed their motivation to work together in the future due to external pressure:

“MINAM and GOREMAD don’t have a good relationship, especially due to MINAM’s detainment of their carbon mapping project. GOREMAD is waiting for MINAM to validate their work (maps). GOREMAD tries to involve MINAM in their activities and invite them to important meetings that they hold so that they can integrate and unite forces. This hasn’t happened much in the past but now due to the Governors Climate Task Fund meetings and getting ready for the COP 20 (Climate Change Conference) there are more plans to work together” (GRRNYMA personal communication 2013).

Tension between the regional and national government was not limited to Madre de Dios. The annual meeting of the Governors’ Climate and Forests (GCF) Task Force, attended by several other Amazonian regional governors, the Ministry of Environment, Regional REDD+ Working Groups, and other Peruvian REDD+ advocates, was held in Puerto Maldonado in September 2013. There, representatives from Loreto proposed creating a stronger alliance among the Amazonian regions to ensure their interests were heard. A regional NGO representative stated:

“We need to align ourselves (referring to Regional REDD+ Working Groups) and come to an agreement… the national position should not be according to Lima but according to the regions.”^14^

The Amazon governor’s regional alliance Consejo Interregional Amazónico (CIAM) committed to organize the regions and help strengthen their knowledge base around related technical issues and capacities for the future implementation of REDD+.

The GCF Task Force also led to at least some important international exchanges for Madre de Dios authorities.^15^The regional government authorities of Madre de Dios and state authorities in Rio Branco, Brazil, exchanged information and proposed an alliance regarding REDD+ and the Verified Carbon Standard Jurisdictional process. This connection between Brazilian GCF Task Force members, Verified Carbon Standard representatives and Madre de Dios’ Regional Government officials was propelled by the Peruvian company Bosques Amazónicos in order to increase the interest and participation of regional officials in the REDD+ process, as well as of local stakeholders in their regional REDD+ project. The BAM employee responsible for this connection alluded to this change in the regional government’s interest and attitude:

“Five years ago, [the Regional Government] was not interested in REDD+ activities and now they are the leaders in the REDD+ Working Group… in addition they are the hosts of the GCF Task Force international meetings in Puerto Maldonado” (2013).

## Discussion: Lessons for Integrated Landscape Governance

Our study suggests that while various actors shaped REDD+ in the region and did improve some land management practices in specific locations, they were not able to shape land-use dynamics more broadly at least in the short term. To date, REDD+ initiatives have had little influence on the primary drivers of deforestation in Madre de Dios. Illegal mining became the main direct driver of deforestation in the region, with inadequate efforts to address it. Key government and private actors continue to support and promote land-use changes from forested areas to agriculture and mining. Agricultural drivers have not been addressed – patches of small papaya plantations proliferate in the landscape. The agriculture offices of the regional government never engaged in the REDD+ debate. Moreover conditions in the La Pampa mining zone continue to deteriorate mostly because REDD+ is not able to be more profitable than gold mining activities. The REDD+ Working Group, as mentioned previously, left some user groups in the region critical of the increasing role of NGOs in Madre de Dios and fearing that NGOs would unduly influence the regional government.

Among other things, REDD+ at the jurisdictional level requires a functioning state authority, which addresses deforestation and degradation drivers, resolves land tenure problems and supports alternative (or sustainable) development pathways. All of these present challenges in the region, and not all of them can be solved locally. The case study of Madre de Dios clearly demonstrates the multilevel nature of the problem and its solutions, as well as the central role of the state, challenging the idea of polycentrism as a workable option (Cole 2011). As one interviewee stated:

“For REDD to work in the region, you need to promote more involvement of political leaders as well as offer them a financial incentive” (Peru Bosques 2013).

This demonstrates the need to have different levels of government interested in promoting REDD. The decentralization process increased local authority over land and forests, but generated confusion, failed to provide technical and financial support and to some extent passed deep-rooted problems on to the regional government. This includes serious problems with mining (which cannot be solved by the region alone; see Gordillo 2014) and land tenure (which requires substantial technical support and funding to be solved, which the regional government has not been able to obtain). Relations between the regional and national government within the REDD+ arena have, however, deteriorated due to the conflict generated by the reference level mapping.

REDD+ resulted, at least temporarily, in some new alliances in Madre de Dios and at broader scales. However, to date there is little evidence of lasting influence on land-use decisions in the region. Starting in 2008, civil society actors took the lead in implementing and promoting REDD+ in Madre de Dios. In some respects they took on the role of government, as suggested in multilevel governance theory (Keck and Sikkink 1998), due to weak and unclear governance structures regarding natural resource use and REDD+. By 2012 and 2013, indigenous federations, along with the Ministry of Environment and the regional government agency of GGRNYMA, began to increase their participation and decision-making power over REDD+, using funding from international donors, thus allowing the REDD+ Working Group to build a strong relationship between governmental and non-governmental actors.

Yet coalition building for transformational change has to go beyond the government and NGOs (Brockhaus et al. 2014; Moeliono et al. 2014; Pham et al. 2014; Sanders et al. 2017; Ravikumar et al. 2015a). Important actors were left out of this coalition. Top-down REDD+ has regularly encountered local resistance (Sanders et al. 2017). Notably, the indigenous federations in the region chose to follow a parallel path, working directly with the central government and donors, rather than risking subordination. Notably, they chose a path that put the issue of territory (indigenous communities, and particularly the formalization of untitled communities) at the core of their REDD+ agenda. Other grassroots actors felt threatened by the power of NGOs, and by their inability to hold them accountable. In a sense, whether true or not, grassroots actors feared that REDD+ “may promote the recentralization of forest governance by allowing a ‘development triangle’ coalition composed of a few powerful government, INGO, and civil society actors to dominate the policy process while simultaneously suppressing the roles and voices of many important stakeholders (Bushley 2014, p.29).” In the case of Madre de Dios, producers feared the influence of NGOs on regional government, as well as their underlying intentions.

Transformational change also requires cross-sectoral collaboration (Brockhaus et al. 2014) and, as suggested by Ravikumar et al. (2015a), the problems seen in this regard at the national level are equally demonstrated by the association of REDD+ initiatives with the weaker environment sector institutions, and the failure to engage with agriculture departments, mining interests or producer associations.

The study found fragmentation of actors across sectors and levels, in spite of some important communication between actors who had not worked together previously. It also found the presence of hybrid actors, which challenge the tendency to view government, civil society and the private sector as clearly defined entities with distinct interests. Such actors form bridges between actor types and levels, as they move between groups, or from regional to national government and promote REDD+ in the region. These individuals provide a counter-argument to the idea that investments in capacity building are lost when people leave their current position—a common complaint about government turnover in particular.

Two outcomes also stand out: first, the early failure of many REDD+ initiatives and second, the loss of the regional elections to an opposing political candidate. By 2014 a new governor, openly pro-mining, was elected in Madre de Dios. He is notably unwilling to work with NGOs, and interaction between NGOs and the government has been very difficult since he took office.

These findings have several implications for efforts to achieve integrated landscape governance. First, although REDD+ embraces more than land-based carbon mitigation, implementation shows that the step toward integrated landscape governance targeting multiple aims (tackling deforestation and forest degradation, sustainable agriculture, livelihood improvement, maintenance of environmental services) across different sectors is not easy to achieve. In principle REDD+ stimulates cross-sectorial approaches by linking forestry and agriculture in project design documents, but in practice agriculture or mining is hardly targeted. Second, although REDD+ creates multi-stakeholder alliances, the implementation in Madre de Dios shows that effective integration of NGOs as well as indigenous people in decision-making is hindered by power imbalances and jurisdictional frictions. Finally, the conclusion that REDD+ implementation requires an effective and functioning state authority and effective communication and collaboration between different levels of government has also implications for integrated landscape governance, suggesting that such state authority is needed to implement the outcomes of multi-stakeholder plans and negotiations.

## Conclusions

As it is home to multiple natural resources, a large mix of actors and interests, and a regional government that recently experienced the reverberations of decentralization, Madre de Dios is a useful case for studying the complex multilevel, multi-sectoral dynamics associated with land use and land-use change, and attempts to change land-use decisions through efforts such as REDD+. This paper contributes to a small body of literature that examines REDD+ at the level of subnational jurisdictions, which are likely to play an increasingly central role in land use, climate mitigation initiatives and landscape governance in the future. Findings indicate that multiple actors shaped REDD+ to some extent, and that REDD+ did bring together key actors in terms of land use and land-use change in the region in new alliances. It is not clear, however, how sustainable those alliances have been, and it does not appear that REDD+ advocates have yet shaped land-use decisions in the region.

Though NGOs have played a very important role in REDD+ and in the region, we find that state and regional governments still play a central role in determining the future of land use and land-use change. For example, in the absence of strong and effective regional regulation, illegal mining continues to be a profitable land-use choice. Without central government intervention, regional governments did not have the means or authority to confront illegal mining, or migration of illegal land users, in the region; it is unlikely that REDD+ projects and carbon payments can offset the price of gold.

Regional governments’ lack of funding and clear authority after decentralization exacerbated problems in resolving overlapping land titles, controlling the influx of migrants in mining and agriculture, and preventing corruption in interactions with the state. Inadequate communication and coordination between regional and national government agencies regarding REDD+ led to an inefficient use of resources to create much needed deforestation and carbon baseline data and caused a temporary rift between actors. This demonstrates the need for better coordination between levels of governance and the inclusion of locally appropriate and acceptable rules.

Advocates for REDD+ in the region also failed to build solid communication with other sectors driving deforestation, such as the regional government agriculture offices, as well as with municipal governments, regional land-user associations (specifically miners and farmers) and indigenous federations. Missing accountabilities between these actors and NGOs fostered tensions based on mistrust of motives and unequal influence over decision-making. We argue that these dynamics represent deep-rooted differences of interest that suggest significant challenges ahead to bring about transformational change. The state still has a central role in influencing outcomes, and institutional solutions need to be found to address the distrust that arises in part from missing accountabilities. The failure to address grassroots concerns may have fostered the change of political party in the regional elections.

Although multi-sectoral and multilevel collaboration and coordination is clearly important to the success of land-based climate mitigation initiatives like REDD+, transformational change needs to be grounded in a clear understanding of interests and incentives and associated power relations. The potential for individuals categorized here as “hybrid actors” to cross categories and help make these alliances and initiatives work deserves further exploration. Transformational change also requires a clear institutional framework for authority and accountability, which highlights the importance of “territory” and the subnational jurisdictions of the state. In terms of integrated landscape governance this means that we need better coordination and collaboration between the multiple actors and stakeholders involved and a better understanding of their interests, in order to balance their inclusion and create sustainable initiatives.

## Footnotes

1 See http://www.cifor.org/gcs/modules/multilevel-governance/legal-reviews/.

2 At the national level, forested land is administrated by a concession system representing a wide variety of economic activities including timber extraction and non-timber forest products such as Brazil nut (*Bertholletia excelsa*) collection, conservation, reforestation, ecotourism, rubber production, and fauna management. The concessionaire is the responsible administrator of the concession for a given period of time.

3 See http://www.cifor.org/gcs/landscapes-governance-peru/.

4 We are not identifying interviewees by name as they did not agree to be cited by name. Instead we will leave their institution name and cite them as anonymous.

5 Fires from slash and burn agriculture are considered a driver of degradation and not deforestation.

6 Peru consists of 25 administrative regions run by regional governments, which are then subdivided into provinces and districts. The region of Madre de Dios has three provinces (Manu, Tahuamanu, and Tambopata), which are composed of eleven districts.

7 In this study, land tenure differs per community member type. Indigenous community members have communal land tenure under one land title, while community members of the religious and non-indigenous settlement of Arca Pacahuara have communal land tenure and practice communal farming. Settlement members and concessionaires hold individual land titles.

8 This individual faced an arrest warrant for defamation and libel, from a judge of the Criminal Court in Loreto. He was accused of committing fraud via carbon-offset projects in the Peruvian Amazon region.

9 In 2010 the Peruvian government issued an emergency decree to place a local office of the Ministry of Environment in Puerto Maldonado due to the escalating problems with illegal mining, and Madre de Dios became the only Region with such an office.

10 Other instances of decentralization occurred with the regional agencies of mining (DREHM) and agriculture (DRA) that were moved from the National Ministry of Agriculture (MINAG) to regional governments, although all but small mining permits are still granted in Lima. In 2010–2011 the regional forestry and wildlife department (DRFFS) was separated from MINAG and the National Institute of Natural Resources (INRENA) and land titling was therefore also transferred from the capital to the regional governments.

11 The PES law passed in June 2014.

12 Verified Carbon Standard certification projects: (1) AIDER’s “Reduction of deforestation and degradation in Tambopata National Reserve and Bahuaja-Sonene National Park within the area of Madre de Dios region”, (2) BAM and FEPROCAMD’s “REDD Project in Brazil nut Concessions in Madre de Dios”, (3) Timber concessions of Maderacre and Maderija’s “Madre de Dios Amazon REDD Project,” which was the first to sell carbon credits. Projects that were in the design process of their project design document were: (4) NGO AIDER & Indigenous community Infierno sustainable forest management and eco-tourism, and (5) FENAMAD Indigenous federation with Bioforest and the Amarakaeiri Reserve. While one initiative was under redevelopment (6) NGO ACCA and the concessionaires of ACOMAT. The remaining nine projects temporarily halted or had been terminated: (7) ACCA Los Amigos conservation area, (8) NGO WWF and the Indigenous Forestry Association of AFIMAD, (9) InkaTierra eco-tourism, (10) NGO Desarrollo Rural Sustentable and the National Parks of Manu-Amarakaeri, (11) BAM company and Timber concession federation FEFOREMAD reforestation project, (12) Indigenous community Belgica and NGO AIDER, (13) NGO Servicios Ecosistemicos, the National Park Manu and the Comunal Reserve Amarakaeri Payments for Environmental Services project, and the two lesser-known projects of (14) Foundation CREES-Piramide, and (15) Forest Carbon Otorongo.

13 The REDD+ Working Group members also noted that their data was of higher quality and thus presented a more accurate reflection of regional vegetation types and carbon stocks than the Ministry of Environment’s model. The Working Group used 14 layers while the Ministry of Environment only used 6, in addition to their higher buy-in due to the inclusive and participatory process in the development of the maps.

14 “Hay que alinearnos y ponernos de acuerdo…. la posición nacional no debe ser según Lima pero según las regiones.”

15 The GCF is composed of members from nine countries.
